# Complete chloroplast genome of *Clematis taeguensis* (Ranunculaceae), an endemic species from South Korea

**DOI:** 10.1080/23802359.2021.1910080

**Published:** 2021-04-22

**Authors:** Beom Kyun Park, Balkrishna Ghimire, Young-Ho Ha, Dong Chan Son, Dong-Kap Kim

**Affiliations:** Division of Forest Biodiversity, Korea National Arboretum, Pocheon, South Korea

**Keywords:** Chloroplast genome, *Clematis taeguensis*, phylogenetic relationship, endemic species

## Abstract

The complete chloroplast (cp) genome sequence of *Clematis taeguensis* Y.N.Lee (Ranunculaceae) was determined to be 159,534 bp in length, consisting of large (79,326 bp) and small (18,338 bp) single-copy regions and a pair of identical inverted repeats (30,935 bp). The genome contains 92 protein-coding genes, 32 tRNA genes, 8 rRNA genes, and 1 pseudogene (*inf*A). Phylogenetic analysis of 19 taxa inferred from the chloroplast genome showed a relationship with *C. taeguensis*, which is also recognized as a species endemic to the Korean Peninsula. The complete cp genome sequence of *C. taeguensis* reported here provides important information for future phylogenetic and evolutionary studies in Ranunculaceae.

The genus *Clematis* L. (Ranunculaceae) comprises about 300 species with worldwide distribution (Wang and Bartholomew 2001; Wang and Li 2005; Kadota 2006; Kim 2017). To date, 13 species have been reported from South Korea (Kim 2017). Among them, four species, including *Clematis taeguensis* Y.N.Lee, are known to be endemic to the Korean Peninsula (Lee 1982, 1996, 2006; Wang 2003a, 2003b; Wang and Li 2005; Chung et al. 2017, Park et al. 2021). This species is easily distinguishable from its closely related taxa by the lack of shoot remains in the winter season, hairy abaxial surface and margin of the sepal, and yellow to brown hairs on the style (Ko and Park 2018; Park et al. 2021). In the present study, we reported the complete chloroplast genome of *C. taeguensis*, and we carried out a phylogenetic analysis to investigate the phylogenetic relationships of *C. taeguensis* with other *Clematis* species.

*Clematis taeguensis* was sampled from Beommul-dong, Daegu-si, South Korea (Voucher specimen: *BK Park, Beommuldong-190719-001*, N35°49′00.2″ E128°39′16.5″). Fresh leaves were silica-dried for DNA extraction. The collected material was stored at the Herbarium of the Korea National Arboretum (KH). DNA was extracted from the silica-dried leaves using a DNeasy Plant Mini Kit (Qiagen, Seoul, Korea) following the manufacturer’ s protocol. The extracted DNA was detected on agarose gel (2%), and high quality gDNA was sequenced using an Illumina MiSeq platform with a 550 bp insert size. Finally, 8,467,638 reads were obtained. The complete chloroplast genome was assembled with Geneious Prime v2020.2.4 (Biotmatters, Auckland, New Zealand). The cp genome was annotated with the Geseq tool (Tillich et al. 2017) and Geneious Prime v2020.2.4 (Biotmatters, Auckland, New Zealand).

The complete cp genome sequence of *C. taeguensis* was 159,534 bp long. The obtained genome sequence was deposited in GenBank (GenBank number MW201572.1). It comprised one large single-copy region (LSC, 79,326 bp), one small single-copy region (SSC, 18,338 bp), and a pair of inverted repeats (IRs, 30,935 bp). The overall GC content of the cp genome was 38%, and in the LSC, SSC, and IRs, it was 36.3%, 31.3%, and 42.1%, respectively. The chloroplast genome contained 136 unique genes, including 92 coding genes, 8 rRNA genes, 32 tRNA genes, and one pseudogene (*inf*A). We found that 25 genes were duplicated in the IR regions.

To infer the phylogenetic relationships among *Clematis* species, the complete cp genome sequences of 19 *Clematis* species were downloaded from GenBank (*Pulsatilla koreana*, *Hepatica asiatica* and *Anemone narcissiflora* species were used as outgroups). A total of 19 species alignments, including *C. taeguensis*, were performed using MAFFT v7.450 (Katoh and Standley 2013; Katoh et al. 2002). The maximum likelihood (ML) bootstrap analysis with 1000 replicates was carried out using RAxML v8.2.11 (Stamatakis 2014). Model selection was performed with JModelTest version 2.1.10 (Darriba et al. 2012) to find the best-fit model (GTR + G + I). The phylogenetic tree showed that *C. taeguensis* was closely related to *C. brachyura* taxa (Figure 1), and plastome was consistent with gene order and content of *C. brachyura*. However, morphologically distinguished from *C. brachyura* because it abaxial and marginal hairs on the sepal, yellowish to brown style hairs, achenes not winged and a persistent style is more than 1.9 cm long (Park et al. 2021). The cp genome sequence of *C. taeguensis* obtained in this study can provide important information for future phylogenetic and evolutionary studies of Ranunculaceae.

**Figure 1.**
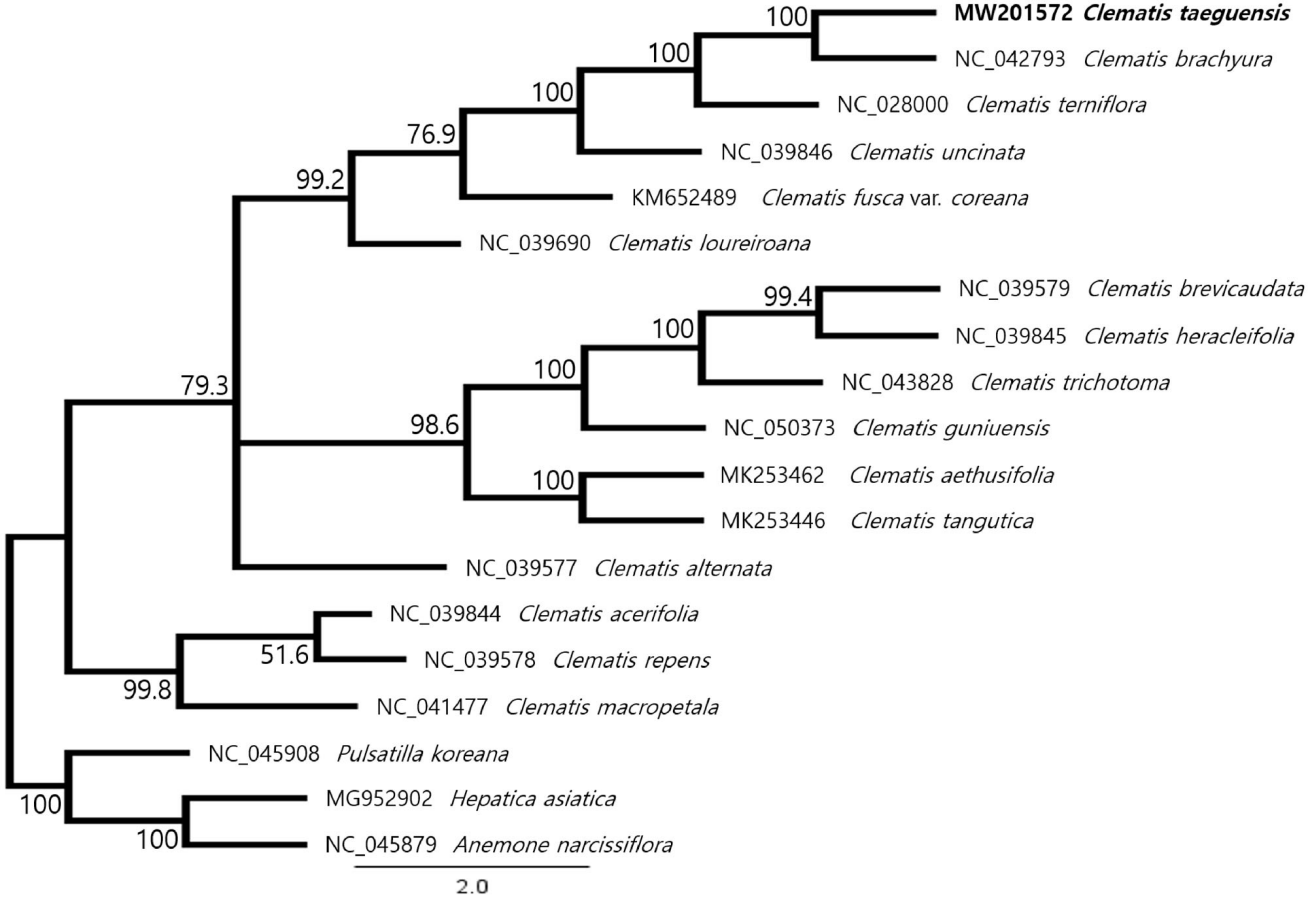
The maximum likelihood phylogenetic tree of *Clematis taeguensis* Y.N.Lee and related taxa based on 92 protein-coding genes. The numbers on each node are the bootstrap support values. The species *Pulsatilla koreana*, *Hepatica asiatica*, and *Anemone narcissiflora* were set as outgroups.

## Data Availability

The data that support the findings of this study are openly available in GenBank of NCBI at https://www.ncbi.nlm.nih.gov, reference number MW201572.1
